# Lipoxins A_4_ and B_4_ inhibit glial cell activation via CXCR3 signaling in acute retinal neuroinflammation

**DOI:** 10.1186/s12974-024-03010-0

**Published:** 2024-01-11

**Authors:** Izhar Livne-Bar, Shubham Maurya, Karsten Gronert, Jeremy M. Sivak

**Affiliations:** 1 Department of Vision Sciences, Donald K Johnson Eye Institute, Krembil Research Institute, University Health Network, Krembil Discovery Tower, 60 Leonard Avenue, Toronto, ON M5T 0S8 Canada; 2 Department of Ophthalmology and Vision Science, University of Toronto School of Medicine, Toronto, Canada; 3 Department of Laboratory Medicine and Pathobiology, University of Toronto School of Medicine, Toronto, Canada; 4 Herbert Wertheim School of Optometry and Vision Science, University of California Berkeley, Berkeley, CA USA; 5 Vision Science Program, University of California Berkeley, Berkeley, CA USA; 6 Infectious Disease and Immunity Program, University of California Berkeley, Berkeley, CA USA

**Keywords:** Lipoxins, Retina, Uveitis, Neuroinflammation, Inflammation resolution, Gliosis, CXCL9, CXCL10, CXCR3

## Abstract

Lipoxins are small lipids that are potent endogenous mediators of systemic inflammation resolution in a variety of diseases. We previously reported that Lipoxins A_4_ and B_4_ (LXA_4_ and LXB_4_) have protective activities against neurodegenerative injury. Yet, lipoxin activities and downstream signaling in neuroinflammatory processes are not well understood. Here, we utilized a model of posterior uveitis induced by lipopolysaccharide endotoxin (LPS), which results in rapid retinal neuroinflammation primarily characterized by activation of resident macroglia (astrocytes and Müller glia), and microglia. Using this model, we observed that each lipoxin reduces acute inner retinal inflammation by affecting endogenous glial responses in a cascading sequence beginning with astrocytes and then microglia, depending on the timing of exposure; prophylactic or therapeutic. Subsequent analyses of retinal cytokines and chemokines revealed inhibition of both CXCL9 (MIG) and CXCL10 (IP10) by each lipoxin, compared to controls, following LPS injection. CXCL9 and CXCL10 are common ligands for the CXCR3 chemokine receptor, which is prominently expressed in inner retinal astrocytes and ganglion cells. We found that CXCR3 inhibition reduces LPS-induced neuroinflammation, while CXCR3 agonism alone induces astrocyte reactivity. Together, these data uncover a novel lipoxin–CXCR3 pathway to promote distinct anti-inflammatory and proresolution cascades in endogenous retinal glia.

**Supplementary Information:**

The online version contains supplementary material available at 10.1186/s12974-024-03010-0.

## Introduction

Neuroinflammation is common to a wide variety of neurological and neurodegenerative diseases, with complex positive and negative effects on tissue damage and repair mechanisms in the central nervous system (CNS). For example, initial inflammatory responses have important roles in fighting infection, debris clearance, and tissue remodeling. However, sustained or extended neuroinflammation can promote increasingly negative consequences, resulting in excessive remodeling and fibrosis, ultimately inducing neurotoxic and neurodegenerative outcomes [1–4]. Many innate neuroinflammatory responses in the CNS are managed by the interacting responses of resident astrocytes and microglial cells, which become rapidly activated upon injury or infection to release cytokines and chemokines, regulate neurotransmission and vascular reactivity, and perform phagocytic functions [1, 5–8]. Accumulating evidence suggests that these roles represent a complex network of activities, rather than simple binary positive and negative phenotypes. Therefore, it is essential to uncover mechanisms that modulate specific components of neuroinflammatory signaling cascades.

The lipoxins, LXA_4_, and LXB_4_, are small endogenous proresolving lipid mediators (SPMs). They belong to a large family of polyunsaturated fatty acid-derived molecules that exhibit potent paracrine or autocrine effects to promote key cellular steps to clear tissue of infiltrating cells and resolve inflammation [9–12]. Lipoxins are derived enzymatically from arachidonic acid by sequential oxygenation [13, 14], and they display a range of established systemic proresolution activities. LXA_4_ binds with high affinity to formyl peptide receptor 2 (ALX/FPR2), to mediate leukocyte recruitment, angiogenesis, and inhibit proinflammatory signaling [13, 15–17]. In comparison, the actions of LXB_4_ can be distinct from LXA_4_ to mediate potent effects on macrophages and monocytes, yet its signaling cascade remains poorly understood [18, 19].

In the CNS, increasing evidence indicates that lipoxin signaling plays a key role in innate neuroinflammatory cell activation and neurodegenerative diseases [20]. Dysregulation of lipoxin synthesis or altered substrate or precursor levels have been reported in both patients samples [21–24] and animal models [21, 25–27] of Alzheimer’s disease (AD). Likewise, dysregulation of this pathway has been observed in other neurodegenerative contexts, including multiple sclerosis (MS) [28], stroke [29, 30], and a variety of ocular diseases [31]. We recently demonstrated that LXA_4_ is reduced in patient samples and attenuates T-cell activities and metabolism in a model of autoimmune uveitis [32]. Previously, we also reported on direct neuroprotective lipoxin actions using models of both retinal and cortical neurodegeneration, and demonstrated that the relevant synthetic circuit and receptor components are expressed in glial cells and neurons [33, 34]. In particular, LXB_4_ consistently exhibited more potent protective activities than LXA_4_ [34]. Thus, in the central nervous system (CNS), lipoxins have potential dual actions to both resolve inflammation and promote neuronal survival. However, the role of these lipoxin actions in neuroinflammation alone has not been clear, particularly their actions with resident glial cells.

Here we profiled the activities of LXA_4_ and LXB_4_ in a model of posterior uveitis; retinal inflammation induced by bacterial lipopolysaccharide (LPS). In characterizing this model we observed responses primarily affecting retinal astrocytes and microglia, which were rapidly and prominently activated following LPS challenge. By treating with lipoxins either prior to- or following challenge, the effects of anti-inflammatory and/or proresolution roles were investigated. In addition, cytokine and chemokine profiling was performed in combination with pharmacologic manipulations in order to identify key signaling related to the chemokine receptor, CXCR3.

## Materials and methods

### LPS-induced uveitis model and drug treatments

All procedures and protocols conformed to the guidelines of the ARVO statement for the use of animals in ophthalmic and vision research, and were approved by the University Health Network Animal Care and Use Committee in accordance with relevant Canadian guidelines and regulations. Briefly, for LPS-induced inflammation, BALB/c mice received an intravitreal injection of 150 ng lipopolysaccharide (LPS) isolated from *E. coli* (Sigma-Millipore) or vehicle, in accordance with previously reported methods [35, 36]. Intravitreal injections were performed as previously described [34, 37]. Briefly, under anesthesia, a needle (30 g) was inserted tangentially into the vitreous cavity and replaced with a Hamilton syringe containing the injection volume. Injections were followed by a 5-s pause before withdrawal. All intravitreal injections were delivered in a 2 μL volume, followed by application of ophthalmic antibiotic ointment (BNP, Vetoquinol). For drug experiments intravitreal injections were performed according to the same method at the indicated times. LXA_4_, LXB_4_ or vehicle (3.5% ETOH) were injected intravitreally at 10 μM (8.8 μg/kg) as previously described [34]. Formulation and dosing of the CXCR3 antagonist AMG487 (Tocris) was based on Ha et al. [38] at 20 mg/kg, i.p., starting 6 h prior to LPS challenge, and then twice daily following LPS. The CXCR3 agonist VUF 11222 (Tocris) was delivered at 500 μM, by two intravitreal injections 24 h apart. Retinas were harvested at 48 h after the 2nd injection.

### Multiplex cytokine analyses

Eyes were injected intravitreally with vehicle, LXB_4_, or LXA_4_ (10 μM each), one hour prior to LPS challenge, as described above. After 24 h retinas were collected and homogenized in 1.5 ml microfuge tubes, snap frozen and stored at -80C. Retinal samples were then submitted to quantitative multiplex array analyses to measure concentrations of a panel of 32 key inflammatory chemokines and cytokines (Eve Technologies) as we have previously reported [39, 40].

### Histology and immunofluorescent staining

Enucleated eyes were fixed in 4% paraformaldehyde for 24 h. Following fixation, the eyes were equilibrated in 30% sucrose, embedded in OCT and cryosectioned at 14 μM thickness. Sections were blocked with 5% donkey serum and probed with primary antibodies to GFAP, Iba1, F4-80, Gr-1, CD3, CD4, CXCR3, CD68, RBPMS and Brn3a, according to our published procedures [37, 39, 41, 42]. The source and details for each antibody are provided in Additional file 1: Table S1. Following primary antibody, sections were washed with PBS-Tween, and incubated with fluorescent-conjugated secondary antibodies (Life-Sciences) and coverslipped with mounting fluorescent medium (DAKO) containing DAPI. A control without primary antibody was assessed on naïve sections in order to identify any unexpected background signals.

### Imaging and analyses

Fluorescent images were acquired on a Nikon Eclipse confocal microscope using Nikon Elements software for image analysis and quantification. Analyses of inflammation markers and RGCs was performed on 8 eyes per treatment. Central retinal sections 150 μM on both sides of the optic nerve were used for quantification. Microglia morphology was used to establish their activation status. Ramified, inner retina, microglia were defined as Iba1-positive cells extending at least one process of 20 μM or longer. Amoeboid microglia had rounded soma but no processes. For morphological assessments stained retinal images were examined at high resolution on a large display and density of amoeboid Iba1-positive cells was counted per 100 μM. For quantification of RGCs Brn3a-positive cells in the central retina were counted and expressed as density per 100 μM along the GCL. Relative intensity was measured by outlining the ganglion cell layer (GCL) and neighboring nerve fiber layer (NFL) and calculating average intensity per μm for the channel in the Nikon Elements software. For each eye, at least 5 central retinal sections were analyzed at the level of the ON as previously described [37].

### Statistical analyses

For all experimental data, ‘*n*’ refers to the number of independent biological replicates. Data were analyzed with either an unpaired *t*-test, or ANOVA with Tukey’s post hoc analyses, as appropriate, using Prism 9.5 software. A *p* value of less than 0.05 was considered significant.

## Results

### Intravitreal LPS induces rapid activation of inner retinal glia

**Fig. 1:**
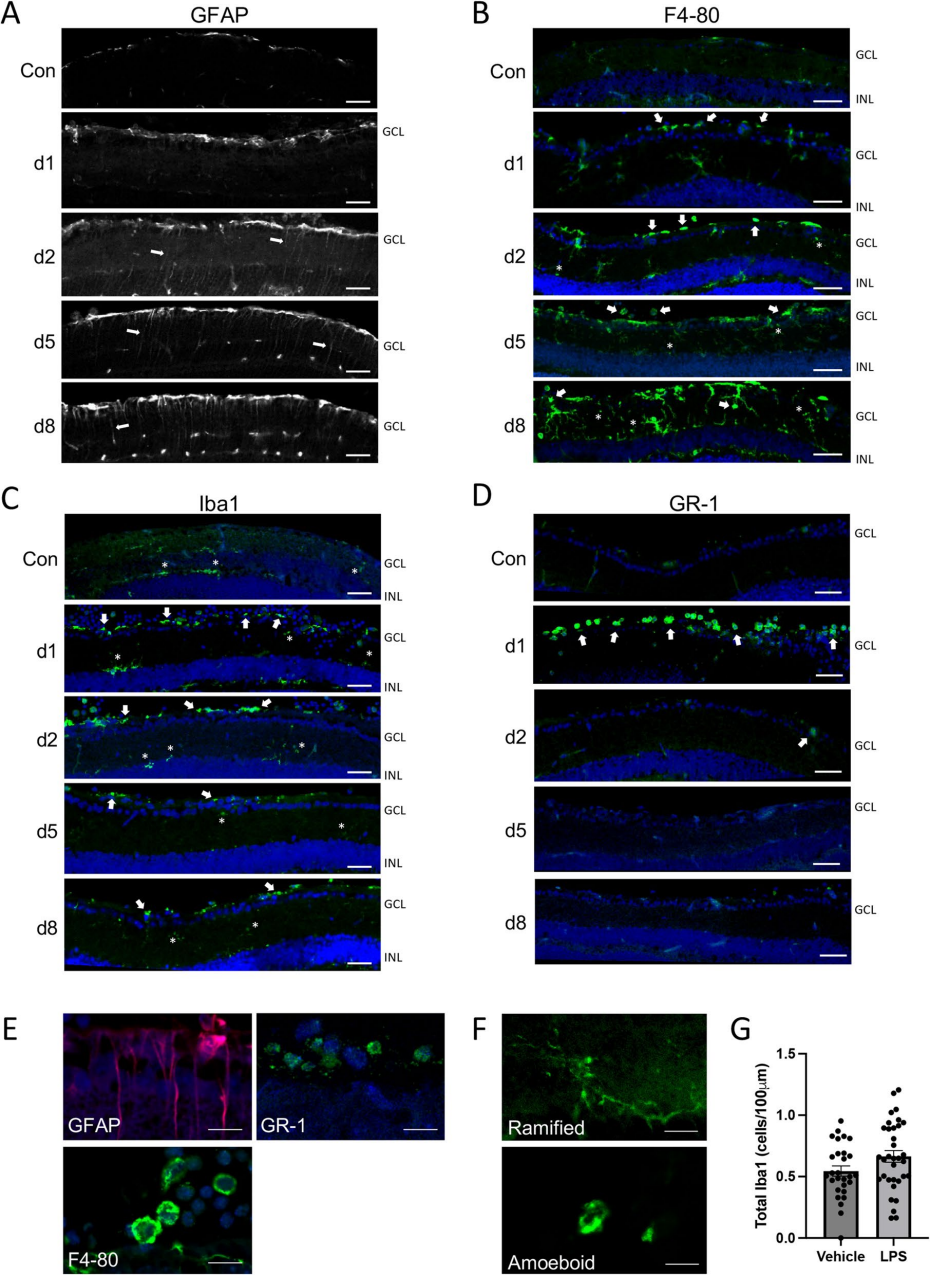
Intravitreal LPS induces rapid activation of inner retinal glial cells. LPS was injected intravitreally and several markers of resident and infiltrating inflammatory cells were monitored over 8 days. **A** Increased Müller cell and astrocyte reactivity in the inner retina was detected with the marker GFAP in glial fibers (arrows) by day 2. **B** Staining with the macrophage marker F4-80 (green) indicated a marked increase of infiltrating cells at the vitreo-retinal interface (arrows) in the inner retina by day 2, as well as activated microglia (asterisks). **C** Iba1 staining for microglial density was generally consistent within the retina across sections (asterisks), but also highlighted the appearance of vitreo-retinal macrophages (arrows). **D** GR-1 positive neutrophils infiltrated into the vitreous by 24 h after LPS (d1) where they accumulated at the inner retinal surface (green; arrows). However, GR-1 positive cells were never substantially observed within the retina, and had largely disappeared by 2 days post LPS injection (d2–d8). **E** Representative higher magnification images of the markers used (scale bars represent 20 µm). **F** Representative retinal Iba-1 stained images of ramified or amoeboid microglial morphology in the LPS-treated group (scale bar represents 20 µm). **G** Quantification of total inner-retina Iba1-positive cells on day 2 shows no significant change in macrophage/microglia numbers in the LPS group compared to vehicle (bars represent SE). (GCL; ganglion cell layer, INL; inner nuclear layer, scale bars of **A**–**D** represent 100 µm)

Models of posterior uveitis by localized intravitreal LPS challenge have been described previously in the literature [35, 36]. However, as this model has not been fully characterized in mice, we performed a time course to profile the inflammatory response and pick optimal study parameters. On day zero 150 ng LPS was administered via intravitreal injection. Eyes were then harvested and processed for sectioning on days 1, 2, 5 and 8. Parallel sections were stained with a panel of antibodies to resident and infiltrating neuroinflammatory markers, including GFAP, F4-80, Iba1, GR-1, CD4 and CD3 (Fig. 1, Additional file 1: Figs. S1, S2). Prominently increased staining intensity was noted for GFAP in retinal astrocytes and Müller glia fibers by day 2 (Fig. 1A). Likewise, staining of infiltrating macrophages with F4-80 at the vitreo-retinal interface was apparent by day 2, along with resident microglia (Fig. 1B). Although overlapping with F4-80, we found that Iba-1 staining was a clearer marker for retinal microglial morphology and remained relatively consistent over the time course (Fig. 1C). Representative high magnification images for each marker are shown in Fig. 1E, F (and larger in Additional file 1: Fig. S1). However, generally figures present a lower magnification image that provides a better indication of the pattern across the retina. The appearance of activated amoeboid microglial cell morphology was apparent in the LPS group (Fig. 1F), and this morphological shift was evaluated quantitatively in subsequent experiments. Corresponding quantification of the retinal total Iba1-positive cell counts showed no significant difference between LPS and vehicle on day 2, suggesting there was no substantial infiltration or proliferation of Iba1 cells (Fig. 1G).

In comparison, systemic immune cell markers had a low profile in the model. Staining for GR-1 indicated rapid initial recruitment of neutrophils into the vitreous that remained localized to the vitreo-retinal interface, did not penetrate into the retina, and largely disappeared by day 2 (Fig. 1D). Notably, we observed no staining of CD4-positive or CD3-positive T cells at any timepoint (Additional file 1: Fig. S2). The numbers of retinal ganglion cells (RGCs) were also assessed to see if there was any neurodegeneration following LPS challenge. No substantial differences were noted at early time points. However, re-assessment at three weeks after challenge revealed an eventual small, but significant loss of BRN3a-positive RGCs after this extended period (Additional file 1: Fig. S3). A qualitative summary of these time course observations is presented in Table 1. Based on these characterizations, we were intrigued to observe such a prominent glial cell response in the model. We chose to use 2–3 day time points in subsequent experiments to evaluate the impact of lipoxins and downstream signals on resident neuroinflammatory cell activation.

**Table 1:** Time course of LPS-induced retinal inflammation cell markers

Marker	Control	Day 1	Day 2	Day 5	Day 8
Müller glia	nd	+	+ + +	+ +	+ +
Amoeboid microglia	nd	+	+ + +	+ +	+
Macrophages	nd	+	+ +	+ +	+ +
Neutrophils	nd	+ +	+	nd	nd
CD4 + T cells	nd	nd	nd	nd	nd
CD3 + T cells	nd	nd	nd	nd	nd

*nd* not detected

### Pretreatment with LXA_4_ or LXB_4_ reduces astrocyte and Müller cell reactivity in the inner retina

**Fig. 2:**
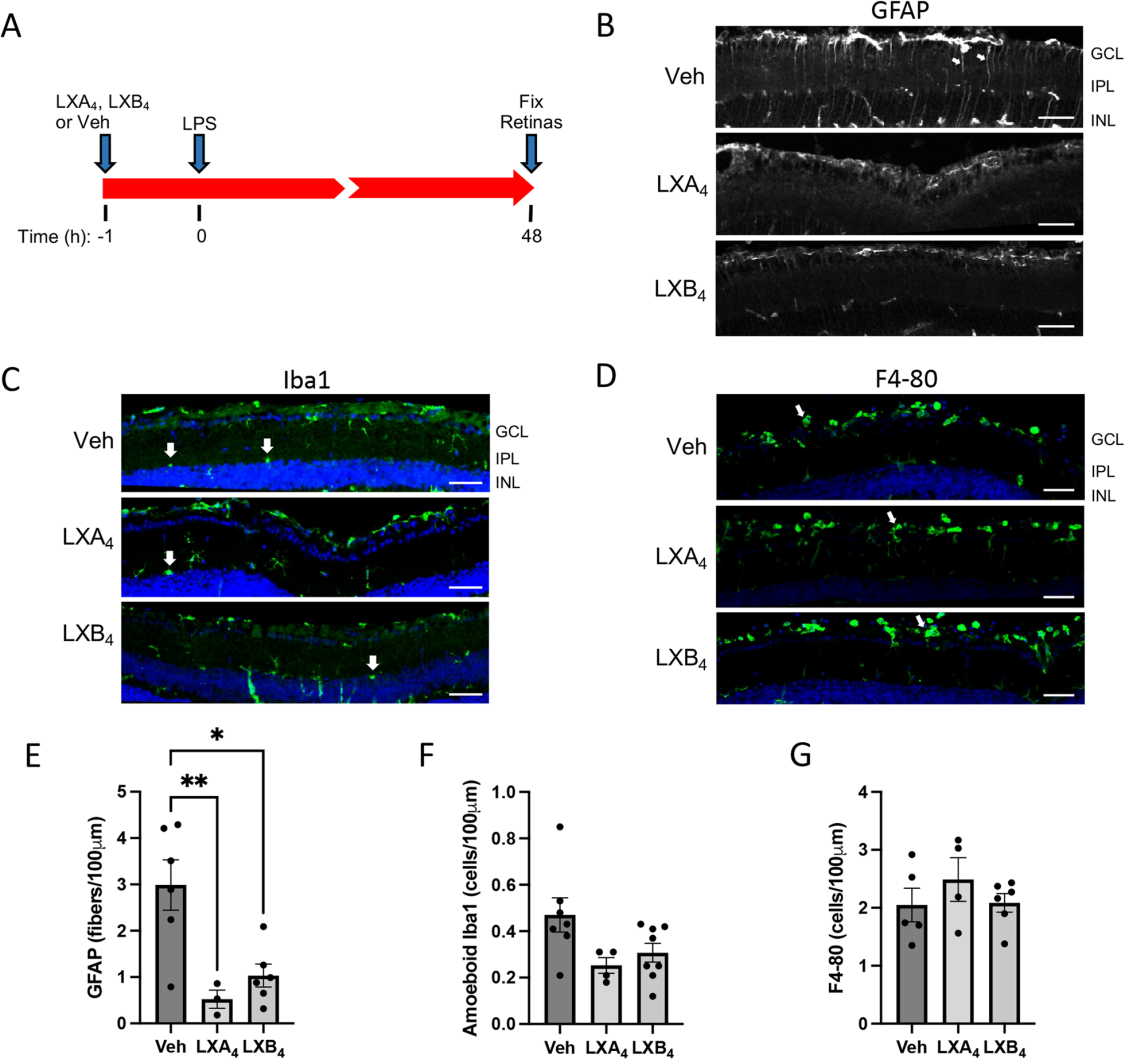
Pretreatment with LXA_4_ or LXB_4_ reduces astrocyte and Müller cell reactivity in the inner retina. **A** Schematic of intravitreal pretreatment and subsequent LPS retinal inflammation assessment at 48 h. **B** Representative images showing pretreatment with LXA_4_ or LXB_4_ strongly reduced GFAP-positive Müller glia fibers in the inner retina (GFAP, arrows). **C** Pretreatment with LXA_4_ or LXB_4_ did not appear to reduce LPS-induced activated microglia (amoeboid Iba1; green, arrows) compared to vehicle control (Veh). **D** Treatment with LXA_4_ or LXB_4_ did not appear to alter the levels of infiltrating macrophages (F4-80: green) at the vitreo-retinal interface (arrows). **E** Corresponding quantification of activated Müller glia in the inner retina expressed as the number of GFAP + processes. LXA_4_ and LXB_4_ pretreatment significantly reduced LPS-activated Müller glia compared with vehicle (Veh, *p < 0.05, **p < 0.01). **F** Quantification of activated (amoeboid) microglia from the inner retina showed no significant reduction with LXA_4_ or LXB_4_ pretreatment compared to vehicle (Veh). **G** Infiltrating macrophages were quantified at the vitreo-retinal interface, showing no significant effect of LXA_4_ and LXB_4_ treatment. (GCL; ganglion cell layer, IPL; inner plexiform layer, INL; inner nuclear layer, scale bars represent 100 µm, graph bars represent SE)

The potential anti-inflammatory activities of LXA_4_ and LXB_4_ were first determined with a pretreatment model, in which 10 μM of either mediator was administered one hour prior to LPS challenge (Fig. 2A). Based on our initial LPS-response time course, after 48 h retinas were fixed and sectioned for staining with our chosen markers, followed by imaging and quantification. At this time point there was a prominent increase in GFAP staining, indicating increased astrocyte and Müller glial reactivity, as expected. However, pretreatment with either LXA_4_ or LXB_4_ had a strong inhibitory effect on this staining (Fig. 2B). Interestingly, quantification showed the activity of LXA_4_ was stronger than LXB_4_ in this regard (Fig. 2E). In comparison, although amoeboid Iba1-positive microglia were present, there was a trend towards inhibition of this activation that did not reach significance (Fig. 2C, F). Finally, there was no apparent effect on infiltrating macrophages at the vitreo-retinal interface (Fig. 2D, G). As a control, we assessed each lipoxin alone with these neuroinflammation markers, and neither had any effect (Additional file 1: Fig. S4). Therefore, the most prominent outcome of lipoxin administration in this pretreatment model was to inhibit astrocyte and Müller cell reactivity.

### Therapeutic treatment with LXA_4_ or LXB_4_ reduces microglial activation in the inner retina

**Fig. 3:**
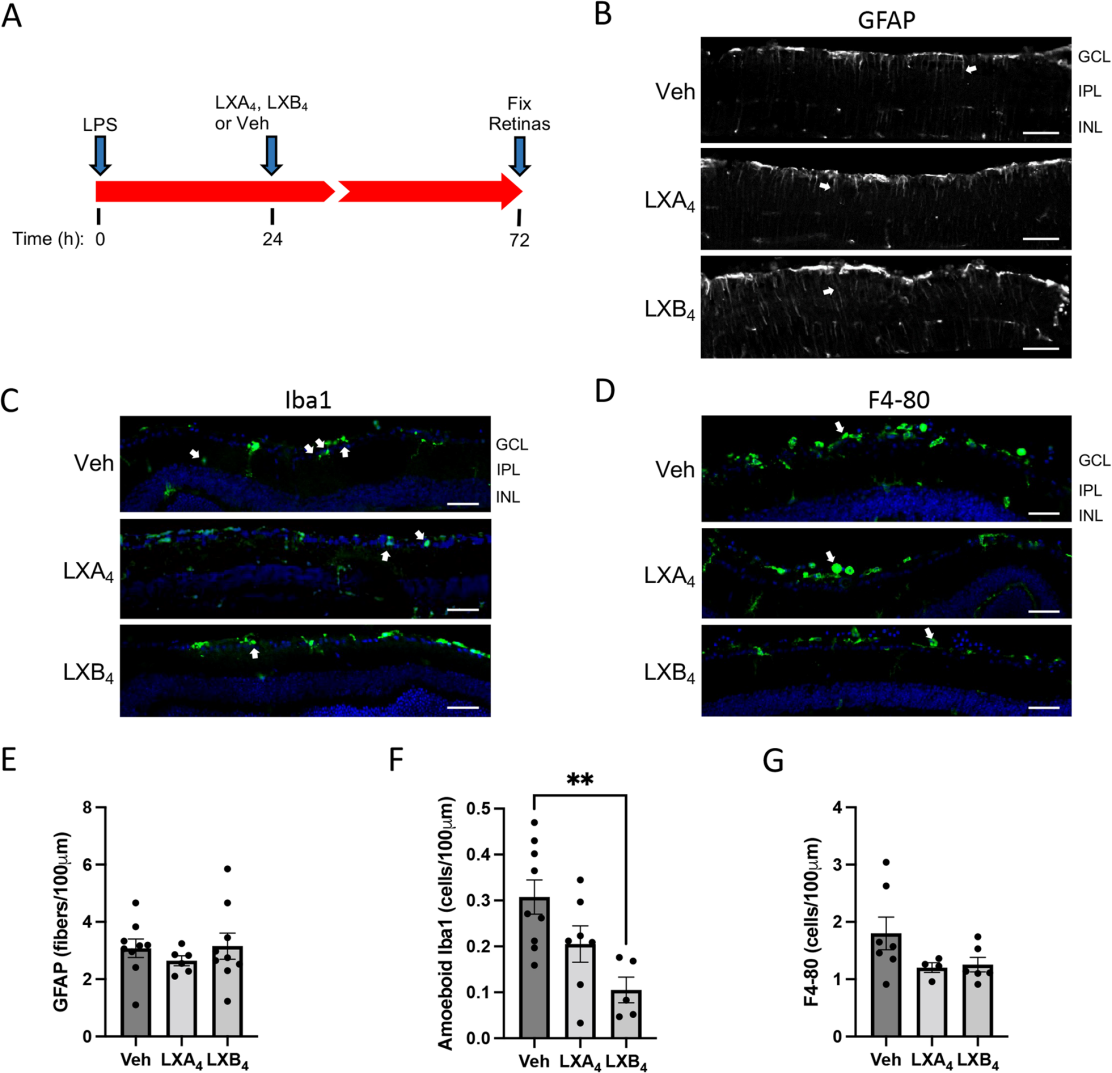
Therapeutic treatment with LXB_4_ reduces microglial activation in the inner retina. **A** Schematic of the LPS-induced retinal inflammation model, subsequent intravitreal treatments and evaluation at 72 h (48 h after lipoxin treatments). **B** Representative images of therapeutic treatment with LXA_4_ or LXB_4_ suggesting little effect on Müller glia activation highlighted by GFAP staining of retinal fibers (arrows). **C** Activated microglia (amoeboid Iba1; green, arrows) in the inner retina were reduced by post-inflammation treatment with LXA_4_ or LXB_4_. **D** Therapeutic treatment with LXA_4_ or LXB_4_ did not substantially reduce the levels of infiltrating macrophages (F4-80; green) accumulating at the vitreal–retinal interface (arrows). **E** Corresponding quantification of activated Müller glia confirmed that LXA_4_ and LXB_4_ post-inflammation treatment had no significant effect compared to Vehicle (Veh). **F** Quantification of activated amoeboid microglia in the inner retina showed that post-inflammation LXB_4_ treatment significantly reduced activated microglia compared to control (**p < 0.01 **G**) Quantification of F4-80 staining confirmed no significant effect of LXA_4_ or LXB_4_ treatment post-inflammation. (GCL; ganglion cell layer, IPL; inner plexiform layer, INL; inner nuclear layer, scale bars represent 100 µm, graph bars represent SE)

By adjusting the timing of administration, we were also able to test the activity of LXA_4_ and LXB_4_ on inflammation resolution in a therapeutic dosing paradigm. In this case, either lipoxin was administered at 24 h after LPS challenge, followed by assessment of the same markers as previously 48 h later (i.e., 72 h from LPS challenge, Fig. 3A). Surprisingly, in this context, the results were distinctly different from the pretreatment experiment. Namely, there was no effect of either lipoxin on retinal astrocyte or Müller cell reactivity (Fig. 3D, E). However, there was instead a strong effect of LXB_4_ treatment on microglial activation, with a similar mild trend induced by LXA_4_ treatment that did not reach significance (Fig. 3B, C). Consistent with the pretreatment model, there was no effect on infiltrating macrophages at the vitreo-retinal interface (Fig. 3F, G). Thus, LXB_4_-mediated inhibition of microglial activation was most prominent when administered following LPS challenge.

### LPS-induced CXCL9/CXCL10 are inhibited by LXA_4_ and LXB_4_ treatment and may interact with the CXCR3 receptor in the inner retina

**Fig. 4:**
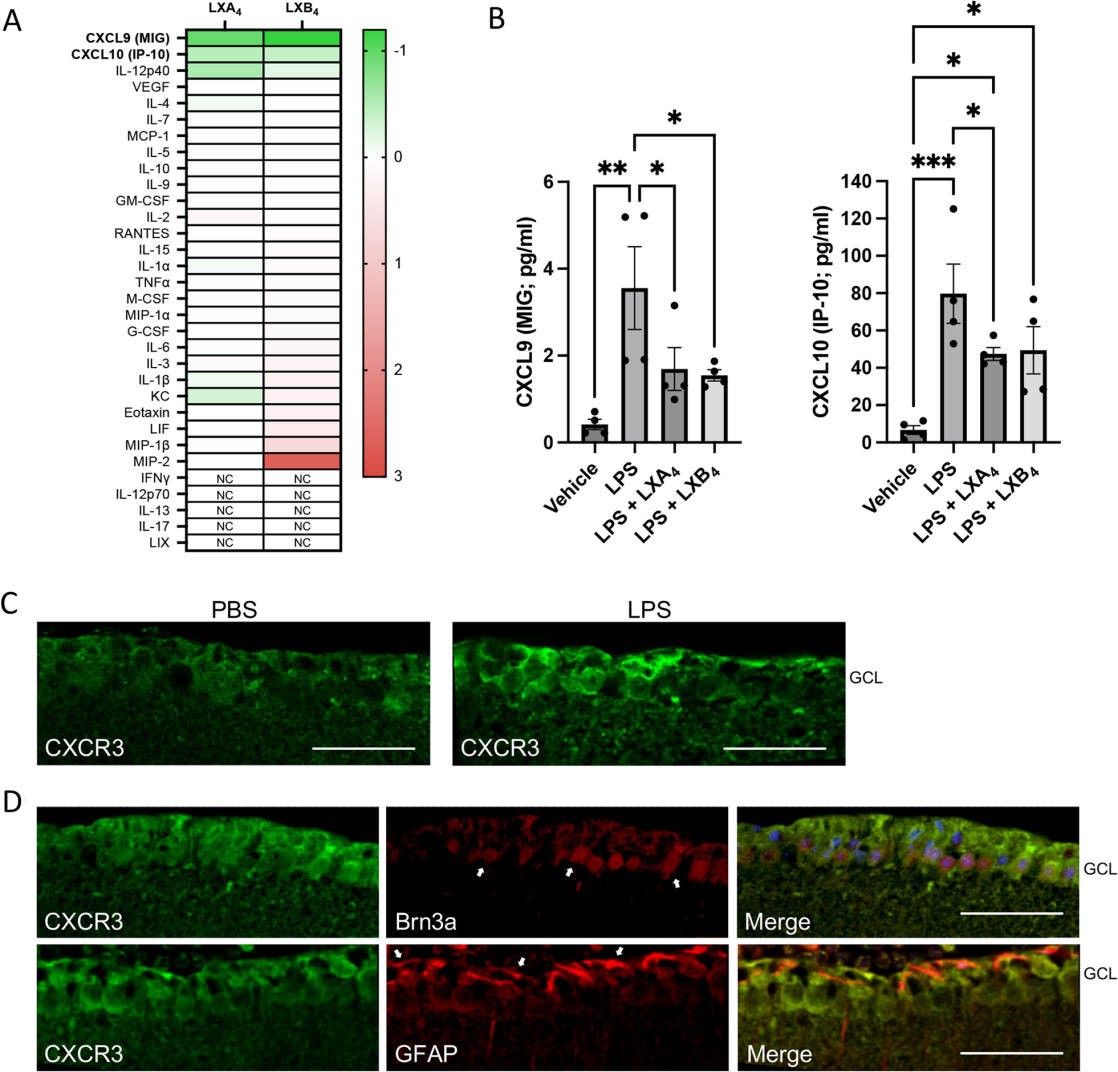
CXCL9/CXCL10 induction is inhibited by LXA_4_ and LXB_4_ treatment and may interact with the CXCR3 receptor in the inner retina. **A** Heatmap of mean multiplex array results for 32 cytokines showing log2 fold inhibition of LPS-induced levels by LXA_4_ or LXB_4_. CXCL9 and CXCL10 were the top hits inhibited by each lipoxin (NC; not calculated due to levels being out of range). **B** Concentrations of CXCL9 and CXCL10 were significantly increased in retinal extracts at 24 h following LPS challenge compared to vehicle. However, eyes pre-treated with LXA_4_ or LXB_4_ showed significantly reduced induction of CXCL9 or CXCL10 levels compared to vehicle (*p < 0.05, **p < 0.01, ***p < 0.005). **C** The shared CXCL9/CXCL10 receptor, CXCR3 (green) is localized to the inner retina (arrows), and appeared induced at two days following LPS challenge. **D** CXCR3 staining partially co-localizes with the RGC marker Brn3a (red, arrows), and **E** CXCR3 (green) also partially co-localizes with GFAP (red, arrows). (Scale bars represent 100 µm, graph bars represent SE)

In order to investigate potential mechanisms underlying lipoxin activities in this model we profiled the levels of 32 retinal cytokines and chemokines induced by LPS challenge in a multiplex array assay. Retinal lysates were assessed at 24 h following LPS challenge in combination with either LXA_4_, LXB_4_ or vehicle pretreatment. The control reference group consisted of retinas injected with PBS only. Following analyses for the strongest and most consistent inhibitory effect, the chemokines CXCL9 (MIG) and CXCL10 (IP-10) stood out prominently as the top two hits (Fig. 4A). Concentrations of each cytokine were strongly and significantly induced by LPS challenge. For CXCL9, the LPS induction was significantly reduced by either LXA_4_ or LXB_4_ treatment (Fig. 4B). In comparison, CXCL10 levels were significantly inhibited by LXA_4_ treatment, while LXB_4_ treatment showed a similar trend that did not reach significance (*p* = 0.06) (Fig. 4B).

Interestingly, both CXCL9 and CXCL10 are common ligands for the CXCR3 chemokine receptor. We therefore probed for CXCR3 localization in control retinas using confocal microscopy. Under control conditions a CXCR3 signal appeared membrane localized, but broadly distributed throughout the inner retina. However, at 48 h following LPS challenge the CXCR3 signal was increased in the ganglion cell layer (Fig. 4C). We tested whether this inner retinal signal overlapped with a marker for nerve fiber layer astrocytes (GFAP) or RGCs (RBPMS), and found partial colocalization for each (Fig. 4D), which might be expected based on the cytoplasmic localization of each cell-type marker, respectively.

### CXCR3 signaling modulates inner retinal neuroinflammation

**Fig. 5:**
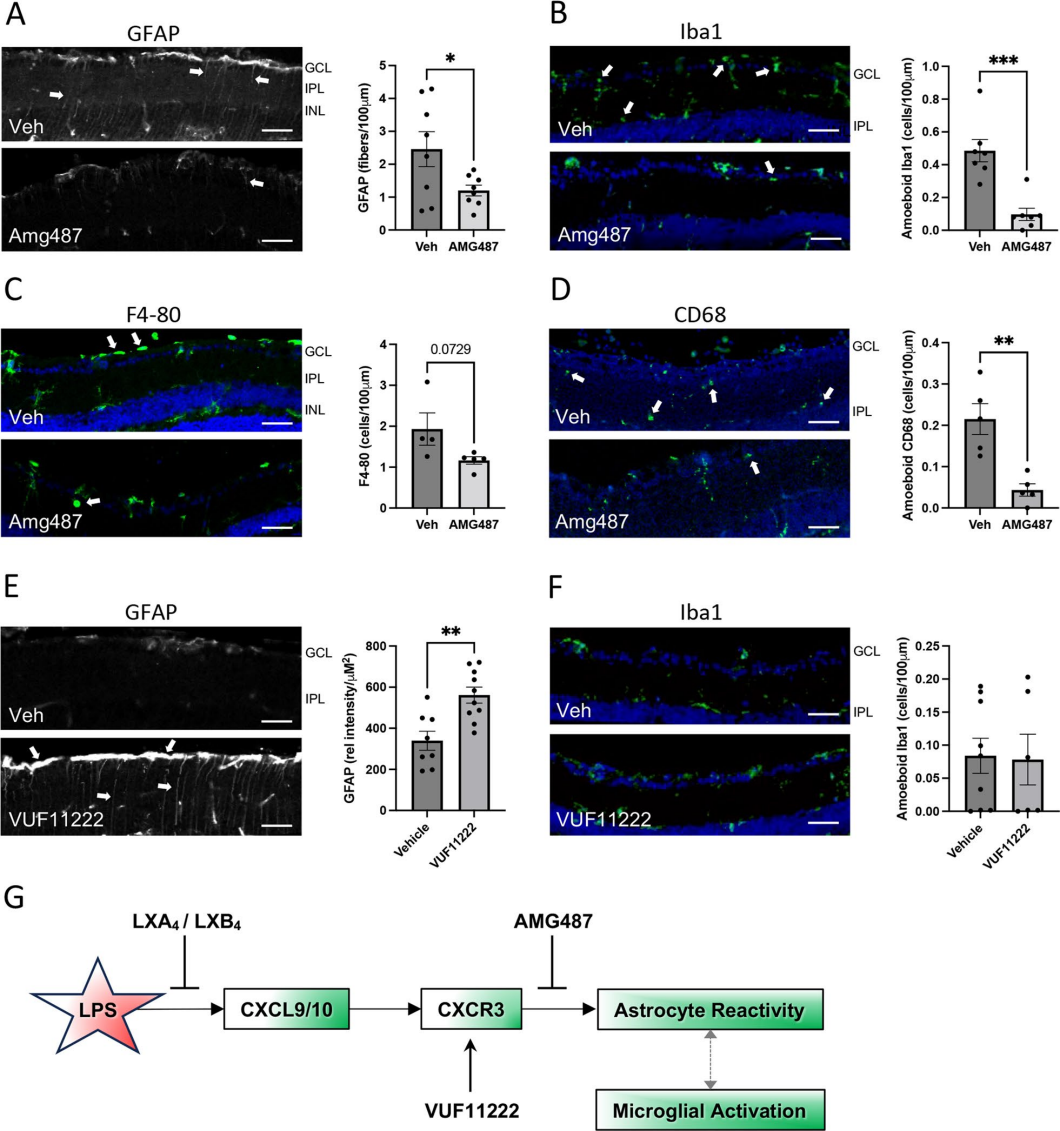
CXCR3 signaling modulates resident retinal inflammation. **A** Representative images indicating treatment with the CXCR3 antagonist AMG487 reduced Müller fiber staining at 48 h following LPS-challenge (green; arrows). Corresponding quantification showed significantly reduced Müller glia activation (*p < 0.05). **B** Treatment with AMG487 reduced amoeboid microglia activation (Iba1; green, arrows). Corresponding quantification of amoeboid microglia showed that AMG487 treatment strongly reduced microglia activation compared to vehicle (***p < 0.0005). **C** AMG487 treatment reduced LPS-induced macrophage infiltration (F4-80; green, arrows), but corresponding quantification showed a trend that did not reach significance. **D** To confirm the microglial results staining was also performed for CD68, which showed a similarly significantly reduced presence of amoeboid cells following AMG487 treatment. **E** In contrast, treatment with the CXCR3 agonist VUF11222 strongly induced astrocyte and Müller glia reactivity (GFAP, arrows). Corresponding quantification showed a significant increase in relative GFAP intensity in the inner retina in the agonist treated group compared to vehicle. **F** VUF11222 treatment had no effect on microglial activation. **G** A cartoon outlining a proposed signaling pathway by which lipoxins inhibit neuroinflammatory responses. (GCL; ganglion cell layer, IPL; inner plexiform layer, INL; inner nuclear layer, bars represent 100 µm, graph bars represent SE)

To confirm that CXCR3 is a necessary component of the LPS injury cascade, we repeated the challenge model while inhibiting its activity. LPS-challenged mice were treated with 20 mg/kg, I.P., of the selective CXCR3 antagonist AMG487 (IC_50_ = 8.2 nM), [43, 44] or vehicle. Administration of AMG487 significantly reduced LPS-induced GFAP in Müller glia (Fig. 5A), and strongly inhibited the appearance of amoeboid microglia (Fig. 5B). AMG487 also reduced vitreal macrophages, although this change did not reach statistical significance (Fig. 5C). As a secondary validation of this strong microglial effect, we also stained for the marker CD68 and quantified amoeboid cells, with similar significant inhibitory effects of AMG487 (Fig. 5D, Additional file 1: Fig. S1). As a control, AMG487 alone had no effect on neuroinflammation markers (Additional file 1: Fig. S4). In contrast, intravitreal treatment with 500 μM, of the CXCR3 agonist, WUF11222 (EC_50_ = 6.1 μM) [45], strongly induced astrocyte and Müller glia GFAP staining (Fig. 5E), but had no effect on amoeboid microglia (Fig. 5F). Therefore, CXCR3 activity is both necessary and sufficient to modulate innate retinal neuroinflammatory responses.

## Discussion

CXCR3 is a G protein-coupled CXC-motif chemokine receptor mediating a variety of inflammatory effects, including chemotaxis, cell survival, and cell division [46, 47]. In turn, CXCR3 activity is induced by binding of the related chemokines CXCL9 and CXCL10, with CXCL11 also binding to a different extracellular site [47–49]. Historically, this signaling has been prominently implicated in T cell recruitment, though we were unable to detect any vitreo-retinal T cell staining in this LPS-uveitis model, at any timepoint. However, CXCL9/10-CXCR3 signaling has also been strongly implicated in the local tissue regulation of microglial recruitment, activation and astrocyte reactivity and neurotoxicity [50–53]. These observations are consistent with our findings, which implicate retinal CXCL9/10-CXCR3 signaling in cascaded activation of resident retinal glia in response to LPS stimulus (Fig. 5F). Accordingly, local tissue activation of this pathway has been reported in pathogenesis of a variety of neuroinflammatory diseases, including Alzheimer’s disease, multiple sclerosis and encephalitis [54–59]. In the retina, CXCL10-CXCR3 expression and function were reported to be required for microglia/monocyte recruitment to the inner retina and to drive neuronal death in pressure-induced ischemia–reperfusion injury [60]. Therefore, the inhibition of CXCL9/10-CXCR3 signaling in resident astrocytes and microglia by lipoxins presents a novel mechanism for targeting this pathway to potentially treat a broad range of neuroinflammatory diseases.

Interestingly, there was a marked difference in lipoxin activities depending on whether they were administered prior to- or following inflammation induction. This observation may reflect time-dependent proresolution lipoxin effects on LPS challenge. Notably, lipoxin pretreatment primarily affected astrocytes and Müller cells, while post-treatment primarily affected microglia. One possible explanation is suggested by the well-described actions of lipoxins on recruitment of neutrophils and macrophages, as well as vascular and tissue permeability [10, 11, 20]. It may be that the difference in timing is influenced by the early and transient presence of these cells impacting the resulting cytokine milieu with respect to pre- or post-treatment. However, as noted we did not detect a robust presence of retinal-infiltrating cells at any time point. Alternatively, it may also be that the resulting tissue signaling is impacted by a suggested cascade of rapid astrocyte reactivity, followed by microglial responses. This interpretation is supported by the strong impact of each lipoxin on pretreatment astrocyte reactivity, and suggests some lipoxin actions occur independent of injury or stress, such as LPS challenge. In addition, the presence of CXCR3 in retinal astrocytes, and the induction of glial reactivity markers by CXCR3 activation is consistent with this idea. Future work will be required to more clearly characterize these cellular responses in detail in different neuroinflammatory contexts.

Previous studies have demonstrated that intravitreal administration of LXA_4_ or analogs can reduce inflammation scores and cell infiltrates when administered simultaneously with LPS challenge in a rat model of *anterior* endotoxin-induced uveitis (EIU), not generally regarded as a neuroinflammation model [61]. Similarly, intravitreal administration of the DHA-derived analog of LXA_4_, resolvin D1 (RvD1), is efficacious when treated one-hour after challenge [62–64]. Recently, we demonstrated that LXA_4_ attenuates T cell-dependent pathology in a model of autoimmune posterior uveitis [32]. However, to date, the roles of lipoxin signaling on neuroinflammatory retinal glia responses have not been well studied. Here, we have profiled the effect of each natural lipoxin on an LPS-induced model of retinal inflammation. Interestingly, our initial characterization suggested this model is prominently driven by endogenous glial reactivity, with little evidence of substantial or sustained infiltration of monocytes, granulocytes, or T cells. Our data demonstrated similar inhibitory activities in this model for both LXA_4_ and LXB_4_ on markers of astrocyte and Müller glia reactivity, microglial activation, and to a limited extent on macrophage infiltration.

Uveitis is broadly defined as intraocular inflammation of the eye’s uveal vascular tract, and is a major cause of vision loss and blindness, worldwide [65, 66]. The inflammatory response can be initiated by either infectious or non-infectious agents, and is broadly categorized as anterior, intermediate, posterior, or panuveitis. Of these, posterior uveitis is the rarest and poorly understood, but has the worst outcomes; often challenging to treat due to the accessibility and vulnerability of inflamed retinal tissue. Corticosteroids and other immunosuppressive therapies can be effective, but early treatment remains critical and can be associated with adverse side effects [67, 68]. In comparison, the roles of lipoxins in posterior uveitis, and their use as potential treatments for retinal inflammation have been relatively poorly studied to date. The efficacy we observed for lipoxin–CXCR3 signaling in a therapeutic setting suggests a novel targeted strategy that may have further benefits for a broad range of diseases associated with retinal inflammation.

### Supplementary Information

**Additional file 1**: **Table S1**. Antibodies Used. **Figure S1**. High magnification images of cell marker staining. A) Representative retinal staining at 1 day following intravitreal LPS challenge, shown at higher magnification to better demonstrate cell morphology of GFAP, F4-80, and GR-1. B) Representative retinal staining for ramified or amoeboid microglial morphology following staining for Iba1 or CD68. (scale bars represent 20 μm). **Figure S2**. LPS challenge did not induce an apparent T-cell response. Representative negative staining for CD4- and CD3-positive T cells in retinal sections at three days following intravitreal LPS treatment showed no signal in the retina. A parallel stained section from a retina three days after exposure to the potent oxidative stressor paraquat (PQ) is presented as a positive control (arrows, scale bars represent 100 μm). **Figure S3**. LPS treatment results in significant RGC loss after three weeks. A) Brn3a staining in retinas over a time course showed no clear reduction in RGC density compared to control until a 21 day time point (d21) after LPS injection (scale bars represent 100 μm). B) Corresponding quantification after 21 days reveals a small, but significant loss of RGCs at three weeks following LPS-induced retinal inflammation (*p <0.05, bars represent SE, GCL; ganglion cell layer). **Figure S4**. LXA_4_, LXB_4_ or Amg487 treatment alone do not induce retinal inflammation markers. Retinal staining for A)GFAP, B) Iba1, or C) F4-80, do not show any difference at two days following treatment, compared to vehicle alone (scale bars represent 100 μm).

## Data Availability

The main data supporting the findings of this study are available within the Article and its Supplemental Information. The raw data materials related to this study are available from the corresponding author upon reasonable request.

## References

[1] Kwon HS, Koh SH (2020). Neuroinflammation in neurodegenerative disorders: the roles of microglia and astrocytes. Transl Neurodegener.

[2] Wyss-Coray T, Mucke L (2002). Inflammation in neurodegenerative disease—a double-edged sword. Neuron.

[3] Alqawlaq S, Flanagan JG, Sivak JM (2019). All roads lead to glaucoma: induced retinal injury cascades contribute to a common neurodegenerative outcome. Exp Eye Res.

[4] Sivak JM (2013). The aging eye: common degenerative mechanisms between the Alzheimer's brain and retinal disease. Invest Ophthalmol Vis Sci.

[5] Liu B, Teschemacher AG, Kasparov S (2017). Astroglia as a cellular target for neuroprotection and treatment of neuro-psychiatric disorders. Glia.

[6] Parpura V, Heneka MT, Montana V, Oliet SH, Schousboe A, Haydon PG, Stout RF, Spray DC, Reichenbach A, Pannicke T, Pekny M, Pekna M, Zorec R, Verkhratsky A (2012). Glial cells in (patho)physiology. J Neurochem.

[7] Garcia-Bermudez MY, Freude KK, Mouhammad ZA, van Wijngaarden P, Martin KK, Kolko M (2021). Glial cells in glaucoma: friends, foes, and potential therapeutic targets. Front Neurol.

[8] Han RT, Kim RD, Molofsky AV, Liddelow SA (2021). Astrocyte-immune cell interactions in physiology and pathology. Immunity.

[9] Gronert K (2008). Lipid autacoids in inflammation and injury responses: a matter of privilege. Mol Interv.

[10] Romano M, Cianci E, Simiele F, Recchiuti A (2015). Lipoxins and aspirin-triggered lipoxins in resolution of inflammation. Eur J Pharmacol.

[11] Serhan CN (2014). Pro-resolving lipid mediators are leads for resolution physiology. Nature.

[12] Perretti M, Dalli J (2022). Resolution pharmacology: focus on pro-resolving annexin a1 and lipid mediators for therapeutic innovation in inflammation. Annu Rev Pharmacol Toxicol.

[13] Ryan A, Godson C (2010). Lipoxins: regulators of resolution. Curr Opin Pharmacol.

[14] Serhan CN, Hamberg M, Samuelsson B (1984). Lipoxins: novel series of biologically active compounds formed from arachidonic acid in human leukocytes. Proc Natl Acad Sci USA.

[15] Cattaneo F, Parisi M, Ammendola R (2013). Distinct signaling cascades elicited by different formyl peptide receptor 2 (FPR2) agonists. Int J Mol Sci.

[16] Maddox JF, Hachicha M, Takano T, Petasis NA, Fokin VV, Serhan CN (1997). Lipoxin A4 stable analogs are potent mimetics that stimulate human monocytes and THP-1 cells via a G-protein-linked lipoxin A4 receptor. J Biol Chem.

[17] Maddox JF, Serhan CN (1996). Lipoxin A4 and B4 are potent stimuli for human monocyte migration and adhesion: selective inactivation by dehydrogenation and reduction. J Exp Med.

[18] Fiore S, Maddox JF, Perez HD, Serhan CN (1994). Identification of a human cDNA encoding a functional high affinity lipoxin A4 receptor. J Exp Med.

[19] Romano M, Maddox JF, Serhan CN (1996). Activation of human monocytes and the acute monocytic leukemia cell line (THP-1) by lipoxins involves unique signaling pathways for lipoxin A4 versus lipoxin B4: evidence for differential Ca^2+^ mobilization. J Immunol.

[20] Kim C, Livne-Bar I, Gronert K, Sivak JM (2020). Fair-weather friends: evidence of lipoxin dysregulation in neurodegeneration. Mol Nutr Food Res.

[21] Firuzi O, Zhuo J, Chinnici CM, Wisniewski T, Pratico D (2008). 5-Lipoxygenase gene disruption reduces amyloid-beta pathology in a mouse model of Alzheimer's disease. FASEB J.

[22] Ikonomovic MD, Abrahamson EE, Uz T, Manev H, Dekosky ST (2008). Increased 5-lipoxygenase immunoreactivity in the hippocampus of patients with Alzheimer's disease. J Histochem Cytochem.

[23] Wang X, Zhu M, Hjorth E, Cortes-Toro V, Eyjolfsdottir H, Graff C, Nennesmo I, Palmblad J, Eriksdotter M, Sambamurti K, Fitzgerald JM, Serhan CN, Granholm AC, Schultzberg M (2015). Resolution of inflammation is altered in Alzheimer's disease. Alzheimers Dement.

[24] Zhu M, Wang X, Hjorth E, Colas RA, Schroeder L, Granholm AC, Serhan CN, Schultzberg M (2016). Pro-resolving lipid mediators improve neuronal survival and increase Abeta42 phagocytosis. Mol Neurobiol.

[25] Chu J, Giannopoulos PF, Ceballos-Diaz C, Golde TE, Pratico D (2012). 5-Lipoxygenase gene transfer worsens memory, amyloid, and tau brain pathologies in a mouse model of Alzheimer disease. Ann Neurol.

[26] Chu J, Li JG, Ceballos-Diaz C, Golde T, Pratico D (2013). The influence of 5-lipoxygenase on Alzheimer's disease-related tau pathology: in vivo and in vitro evidence. Biol Psychiatry.

[27] Dunn HC, Ager RR, Baglietto-Vargas D, Cheng D, Kitazawa M, Cribbs DH, Medeiros R (2015). Restoration of lipoxin A4 signaling reduces Alzheimer's disease-like pathology in the 3xTg-AD mouse model. J Alzheimer's Disease JAD.

[28] Pruss H, Rosche B, Sullivan AB, Brommer B, Wengert O, Gronert K, Schwab JM (2013). Proresolution lipid mediators in multiple sclerosis—differential, disease severity-dependent synthesis—a clinical pilot trial. PLoS ONE.

[29] Vital SA, Becker F, Holloway PM, Russell J, Perretti M, Granger DN, Gavins FN (2016). Formyl-peptide receptor 2/3/lipoxin A4 receptor regulates neutrophil-platelet aggregation and attenuates cerebral inflammation: impact for therapy in cardiovascular disease. Circulation.

[30] Yigitkanli K, Pekcec A, Karatas H, Pallast S, Mandeville E, Joshi N, Smirnova N, Gazaryan I, Ratan RR, Witztum JL, Montaner J, Holman TR, Lo EH, van Leyen K (2013). Inhibition of 12/15-lipoxygenase as therapeutic strategy to treat stroke. Ann Neurol.

[31] Wei J, Gronert K (2017). The role of pro-resolving lipid mediators in ocular diseases. Mol Aspects Med.

[32] Wei J, Mattapallil MJ, Horai R, Jittayasothorn Y, Modi AP, Sen HN, Gronert K, Caspi RR. A novel role for lipoxin A(4) in driving a lymph node-eye axis that controls autoimmunity to the neuroretina. Elife. 2020;9. 10.7554/eLife.51102 PMC7064344 32118582

[33] Lee FC, Brown CE, Nielsen AJ, Kim C, Livne-Bar I, Parsons PJ, Boldron C, Autelitano F, Weaver DF, Sivak JM, Reed MA (2022). A stereocontrolled total synthesis of lipoxin B4 and its biological activity as a pro-resolving lipid mediator of neuroinflammation. Chemistry.

[34] Livne-Bar I, Wei J, Liu HH, Alqawlaq S, Won GJ, Tuccitto A, Gronert K, Flanagan JG, Sivak JM (2017). Astrocyte-derived lipoxins A4 and B4 promote neuroprotection from acute and chronic injury. J Clin Investig.

[35] Lafreniere JD, Toguri JT, Gupta RR, Samad A, O'Brien DM, Dickinson J, Cruess A, Kelly MEM, Seamone ME (2019). Effects of intravitreal bevacizumab in Gram-positive and Gram-negative models of ocular inflammation. Clin Exp Ophthalmol.

[36] Toguri JT, Lehmann C, Laprairie RB, Szczesniak AM, Zhou J, Denovan-Wright EM, Kelly ME (2014). Anti-inflammatory effects of cannabinoid CB(2) receptor activation in endotoxin-induced uveitis. Br J Pharmacol.

[37] Livne-Bar I, Lam S, Chan D, Guo X, Askar I, Nahirnyj A, Flanagan JG, Sivak JM (2016). Pharmacologic inhibition of reactive gliosis blocks TNF-alpha-mediated neuronal apoptosis. Cell Death Dis.

[38] Ha Y, Liu H, Zhu S, Yi P, Liu W, Nathanson J, Kayed R, Loucas B, Sun J, Frishman LJ, Motamedi M, Zhang W (2017). Critical role of the CXCL10/C-X-C chemokine receptor 3 axis in promoting leukocyte recruitment and neuronal injury during traumatic optic neuropathy induced by optic nerve crush. Am J Pathol.

[39] Mathew DJ, Livne-Bar I, Sivak JM (2021). An inducible rodent glaucoma model that exhibits gradual sustained increase in intraocular pressure with distinct inner retina and optic nerve inflammation. Sci Rep.

[40] Nahirnyj A, Livne-Bar I, Guo X, Sivak JM (2013). ROS detoxification and proinflammatory cytokines are linked by p38 MAPK signaling in a model of mature astrocyte activation. PLoS ONE.

[41] Guo X, Jiang Q, Tuccitto A, Chan D, Alqawlaq S, Won GJ, Sival JM (2018). The AMPK-PGC-1α signaling axis regulates the astrocyte glutathione system to protect against oxidative and metabolic injury. Neurobiol Dis.

[42] Lin A, Guo X, Inman RD, Sivak JM (2015). Ocular inflammation in HLA-B27 transgenic mice reveals a potential role for MHC class I in corneal immune privilege. Mol Vis.

[43] Johnson M, Li AR, Liu J, Fu Z, Zhu L, Miao S, Wang X, Xu Q, Huang A, Marcus A, Xu F, Ebsworth K, Sablan E, Danao J, Kumer J, Dairaghi D, Lawrence C, Sullivan T, Tonn G, Schall T, Collins T, Medina J (2007). Discovery and optimization of a series of quinazolinone-derived antagonists of CXCR3. Bioorg Med Chem Lett.

[44] Walser TC, Rifat S, Ma X, Kundu N, Ward C, Goloubeva O, Johnson MG, Medina JC, Collins TL, Fulton AM (2006). Antagonism of CXCR3 inhibits lung metastasis in a murine model of metastatic breast cancer. Can Res.

[45] Wijtmans M, Scholten DJ, Roumen L, Canals M, Custers H, Glas M, Vreeker MC, de Kanter FJ, de Graaf C, Smit MJ, de Esch IJ, Leurs R (2012). Chemical subtleties in small-molecule modulation of peptide receptor function: the case of CXCR3 biaryl-type ligands. J Med Chem.

[46] Muller M, Carter S, Hofer MJ, Campbell IL (2010). Review: the chemokine receptor CXCR3 and its ligands CXCL9, CXCL10 and CXCL11 in neuroimmunity—a tale of conflict and conundrum. Neuropathol Appl Neurobiol.

[47] Koper OM, Kaminska J, Sawicki K, Kemona H (2018). CXCL9, CXCL10, CXCL11, and their receptor (CXCR3) in neuroinflammation and neurodegeneration. Adv Clin Exp Med.

[48] Clark-Lewis I, Mattioli I, Gong JH, Loetscher P (2003). Structure-function relationship between the human chemokine receptor CXCR3 and its ligands. J Biol Chem.

[49] Lazzeri E, Romagnani P (2005). CXCR3-binding chemokines: novel multifunctional therapeutic targets. Curr Drug Targets Immune Endocr Metabol Disord.

[50] Li H, Gang Z, Yuling H, Luokun X, Jie X, Hao L, Li W, Chunsong H, Junyan L, Mingshen J, Youxin J, Feili G, Boquan J, Jinquan T (2006). Different neurotropic pathogens elicit neurotoxic CCR9- or neurosupportive CXCR3-expressing microglia. J Immunol.

[51] Sui Y, Stehno-Bittel L, Li S, Loganathan R, Dhillon NK, Pinson D, Nath A, Kolson D, Narayan O, Buch S (2006). CXCL10-induced cell death in neurons: role of calcium dysregulation. Eur J Neurosci.

[52] Biber K, Dijkstra I, Trebst C, De Groot CJ, Ransohoff RM, Boddeke HW (2002). Functional expression of CXCR3 in cultured mouse and human astrocytes and microglia. Neuroscience.

[53] Goldberg SH, van der Meer P, Hesselgesser J, Jaffer S, Kolson DL, Albright AV, Gonzalez-Scarano F, Lavi E (2001). CXCR3 expression in human central nervous system diseases. Neuropathol Appl Neurobiol.

[54] Koper OM, Kaminska J, Grygorczuk S, Zajkowska J, Kemona H (2018). CXCL9 concentrations in cerebrospinal fluid and serum of patients with tick-borne encephalitis. Arch Med Sci.

[55] Salmaggi A, Gelati M, Dufour A, Corsini E, Pagano S, Baccalini R, Ferrero E, Scabini S, Silei V, Ciusani E, De Rossi M (2002). Expression and modulation of IFN-gamma-inducible chemokines (IP-10, Mig, and I-TAC) in human brain endothelium and astrocytes: possible relevance for the immune invasion of the central nervous system and the pathogenesis of multiple sclerosis. J Interferon Cytokine Res.

[56] Simpson JE, Newcombe J, Cuzner ML, Woodroofe MN (2000). Expression of the interferon-gamma-inducible chemokines IP-10 and Mig and their receptor, CXCR3, in multiple sclerosis lesions. Neuropathol Appl Neurobiol.

[57] Xia MQ, Bacskai BJ, Knowles RB, Qin SX, Hyman BT (2000). Expression of the chemokine receptor CXCR3 on neurons and the elevated expression of its ligand IP-10 in reactive astrocytes: in vitro ERK1/2 activation and role in Alzheimer's disease. J Neuroimmunol.

[58] Satarkar D, Patra C (2022). Evolution, expression and functional analysis of CXCR3 in neuronal and cardiovascular diseases: a narrative review. Front Cell Dev Biol.

[59] Zhou YQ, Liu DQ, Chen SP, Sun J, Zhou XR, Xing C, Ye DW, Tian YK (2019). The role of CXCR3 in neurological diseases. Curr Neuropharmacol.

[60] Ha Y, Liu H, Xu Z, Yokota H, Narayanan SP, Lemtalsi T, Smith SB, Caldwell RW, Caldwell RB, Zhang W (2015). Endoplasmic reticulum stress-regulated CXCR3 pathway mediates inflammation and neuronal injury in acute glaucoma. Cell Death Dis.

[61] Karim MJ, Bhattacherjee P, Biswas S, Paterson CA (2009). Anti-inflammatory effects of lipoxins on lipopolysaccharide-induced uveitis in rats. J Ocul Pharmacol Ther.

[62] Rossi S, Di Filippo C, Gesualdo C, Potenza N, Russo A, Trotta MC, Zippo MV, Maisto R, Ferraraccio F, Simonelli F, D'Amico M (2015). Protection from endotoxic uveitis by intravitreal Resolvin D1: involvement of lymphocytes, miRNAs, ubiquitin-proteasome, and M1/M2 macrophages. Mediators Inflamm.

[63] Rossi S, Di Filippo C, Gesualdo C, Testa F, Trotta MC, Maisto R, Ferraro B, Ferraraccio F, Accardo M, Simonelli F, D'Amico M (2015). Interplay between intravitreal RvD1 and local endogenous sirtuin-1 in the protection from endotoxin-induced uveitis in rats. Mediators Inflamm.

[64] Settimio R, Clara DF, Franca F, Francesca S, Michele D (2012). Resolvin D1 reduces the immunoinflammatory response of the rat eye following uveitis. Mediators Inflamm.

[65] Durrani OM, Tehrani NN, Marr JE, Moradi P, Stavrou P, Murray PI (2004). Degree, duration, and causes of visual loss in uveitis. Br J Ophthalmol.

[66] Tsirouki T, Dastiridou A, Symeonidis C, Tounakaki O, Brazitikou I, Kalogeropoulos C, Androudi S (2018). A focus on the epidemiology of uveitis. Ocul Immunol Inflamm.

[67] Tomkins-Netzer O, Talat L, Bar A, Lula A, Taylor SR, Joshi L, Lightman S (2014). Long-term clinical outcome and causes of vision loss in patients with uveitis. Ophthalmology.

[68] Levy-Clarke G, Jabs DA, Read RW, Rosenbaum JT, Vitale A, Van Gelder RN (2014). Expert panel recommendations for the use of anti-tumor necrosis factor biologic agents in patients with ocular inflammatory disorders. Ophthalmology.

