# Supplementary material for: Lipoxins A4 and B4 inhibit glial cell activation via CXCR3 signaling in acute retinal neuroinflammation
Source: J Neuroinflammation. 2024 Jan 11;21:18. doi: 10.1186/s12974-024-03010-0 (PMC10782675; doi:10.1186/s12974-024-03010-0)
Supplement: Supplementary file 1 — Additional file 1: Table S1. Antibodies Used. Figure S1. High magnification images of cell marker staining. A) Representative retinal staining at 1 day following intravitreal LPS challenge, shown at higher magnification to better demonstrate cell morphology of GFAP, F4-80, and GR-1. B) Representative retinal staining for ramified or amoeboid microglial morphology following staining for Iba1 or CD68. (scale bars represent 20 μm). Figure S2. LPS challenge did not induce an apparent T-cell response. Representative negative staining for CD4- and CD3-positive T cells in retinal sections at three days following intravitreal LPS treatment showed no signal in the retina. A parallel stained section from a retina three days after exposure to the potent oxidative stressor paraquat (PQ) is presented as a positive control (arrows, scale bars represent 100 μm). Figure S3. LPS treatment results in significant RGC loss after three weeks. A) Brn3a staining in retinas over a time course showed no clear reduction in RGC density compared to control until a 21 day time point (d21) after LPS injection (scale bars represent 100 μm). B) Corresponding quantification after 21 days reveals a small, but significant loss of RGCs at three weeks following LPS-induced retinal inflammation (*p <0.05, bars represent SE, GCL; ganglion cell layer). Figure S4. LXA4, LXB4 or Amg487 treatment alone do not induce retinal inflammation markers. Retinal staining for A)GFAP, B) Iba1, or C) F4-80, do not show any difference at two days following treatment, compared to vehicle alone (scale bars represent 100 μm). [file 12974_2024_3010_MOESM1_ESM.pdf]

Table S1 . Antibodies Used

Marker	Source	Catalogue #	Dilution	Species/Type*
**GFAP**	Sigma	G3893	1:220	Mouse/MAB
**Iba-1**	Novus	NB100-1028	1:250	Goat/Poly
**Cd68**	Biolegend	137001	1:100	Rat/MAB
**F4-80**	Biolegend	122601	1:100	Rat/MAB
**GR-1**	Biolegend	108403	1:100	Rat/MAB
**CD4**	Invitrogen	140040-82	1:80	Mouse/MAB
**CD3**	Invitrogen	90582	1:80	Rabbit/MAB
**CxcR3**	Novus	41250	1:100	Rabbit/Poly
**Brn3a**	Santa Cruz	31984	1:300	Goat/Poly
**Rbpms**	PhosphoSolutions	1830	1:300	Rabbit/Poly

**Poly; polyclonal antibody, MAB; monoclonal antibody*

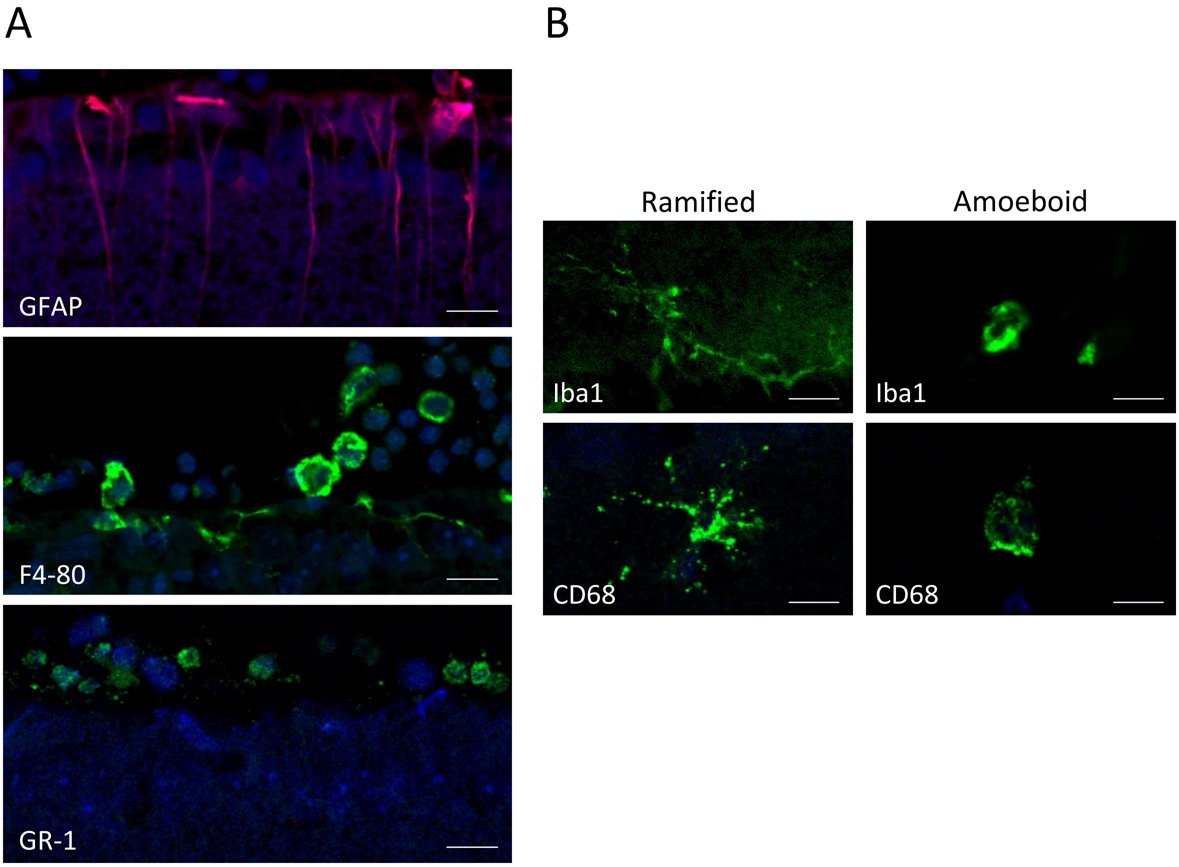

Figure S1. High magnification images of cell marker staining. A) Representative retinal staining at 1 day following intravitreal LPS challenge, shown at higher magnification to better demonstrate cell morphology of GFAP, F4-80, and GR-1. B) Representative retinal staining for ramified or amoeboid microglial morphology following staining for Iba1 or CD68. (scale bars represent 20 µm).

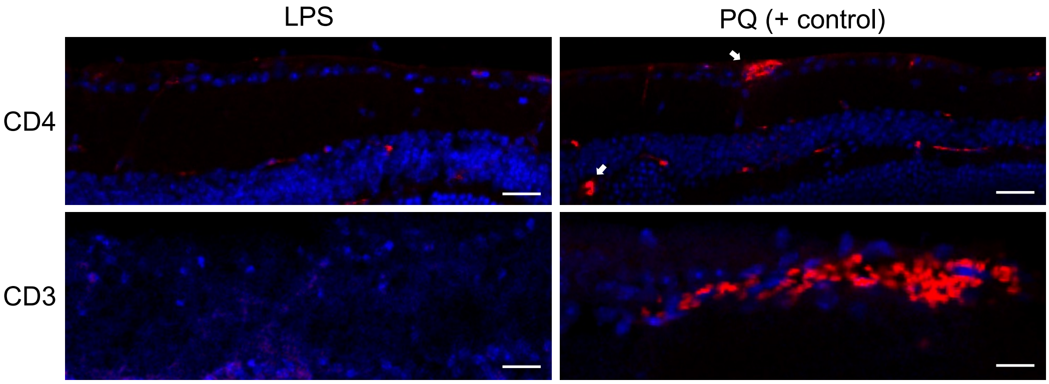

Figure S2. LPS challenge did not induce an apparent T-cell response. Representative negative staining for CD4- and CD3-positive T cells in retinal sections at three days following intravitreal LPS treatment showed no signal in the retina. A parallel stained section from a retina three days after exposure to the potent oxidative stressor paraquat (PQ) is presented as a positive control (arrows, scale bars represent 100 µm).

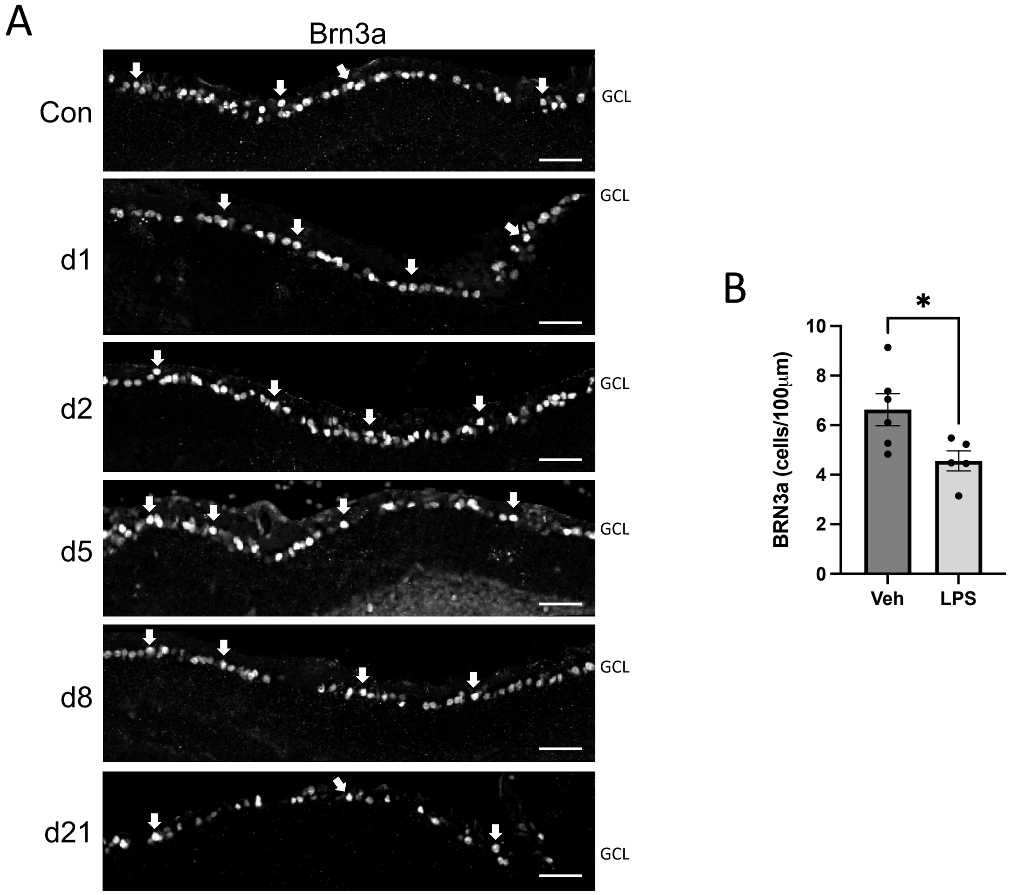

Figure S3. LPS treatment results in significant RGC loss after three weeks. A) Brn3a staining in retinas over a time course showed no clear reduction in RGC density compared to control until a 21 day time point (d21) after LPS injection (scale bars represent 100 μm). B) Corresponding quantification after 21 days reveals a small, but significant loss of RGCs at three weeks following LPS induced retinal inflammation (*p <0.05, bars represent SE, GCL; ganglion cell layer).

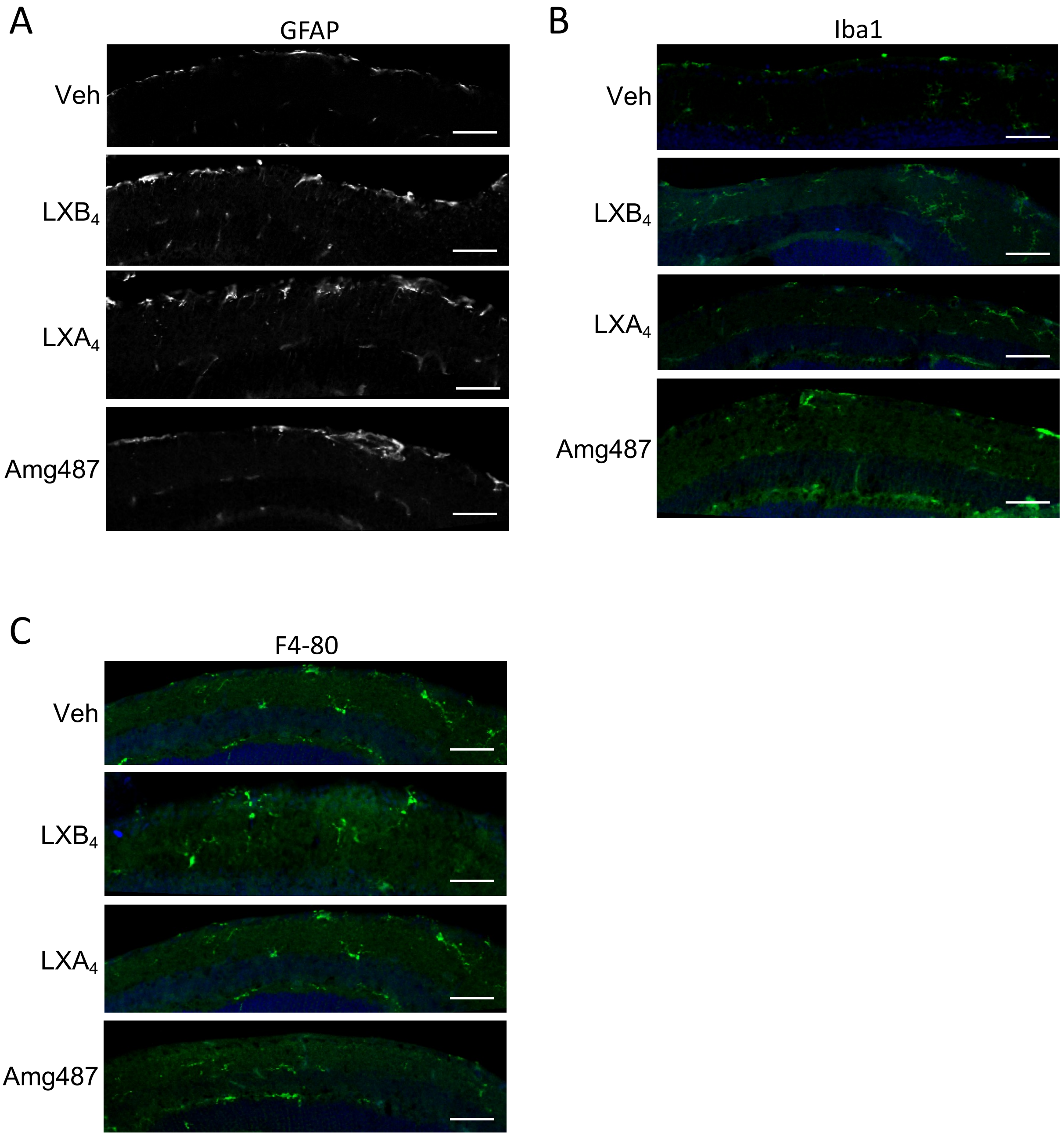

Figure S4. LXA_4, LXB_4 or Amg487 treatment alone do not induce retinal inflammation markers. Retinal staining for A)GFAP, B) Iba1, or C) F4-80, do not show any difference at two days following treatment, compared to vehicle alone (scale bars represent 100 µm).
